# Isolation and Identification of Some Probiotic Bacteria and Their Potential Role in Improving Immune Response and Resistance of Nile Tilapia *(Oreochromis niloticus*) in Comparison with a Commercial Product

**DOI:** 10.1155/2020/8865456

**Published:** 2020-07-17

**Authors:** Mosleh M. Abomughaid

**Affiliations:** Medical Laboratory Sciences Department, College of Applied Medical Sciences, University of Bisha, Bisha, Saudi Arabia

## Abstract

This work aimed to retrieve a field isolate of probiotic from Nile tilapia *(Oreochromis niloticus*) and compare the obtained results with a commercial probiotic product through experimental studies. The study was conducted on 250 Nile tilapia. Ten fish were used to isolate the probiotic strain. Two isolates showed an in vitro inhibitory effect against pathogenic *A. hydrophila*. The isolate with the largest zone was identified by PCR. Sixty fish were used to test the safety of a potential probiotic. One hundred and eighty fish were used in a two-month feeding experiment. Fish were divided into 3 groups, group (1): the control, group (2): fed on potential probiotics, and group (3): fed on commercial probiotic (Organic Green™). The effects of tested products on the immune response were recorded in all groups. After one and two months of feeding experiment, blood and nonspecific immune parameters were evaluated. Disease resistance against *Aeromonas hydrophila* was evaluated through challenge experiment. The histopathology of the treated groups was fully recorded in comparison with the control group. The potential probiotic based on the in vitro antimicrobial activity test was identified as *P. putida* using routine and gel electrophoresis and 16S rRNA sequencing. During the first and the second month of experiment, there was a highly significant increase in the survival percent of the experimental fish in both treated groups with probiotics. In the first phase of the experiment, a significant increase in the haematocrit values and NBT, lysozyme activity, and phagocytic activity was seen in all treated groups in comparison with the control. The increase in the TLC was significant in the group fed with *P. putida* in comparison with the control group. In the second phase, a nonsignificant increase in the hematocrit values and significant increases in the NBT and phagocytic index were seen in *P. putida* and organic green groups in comparison with the control group. The TLC and DLC revealed nonsignificant changes in the treated groups in comparison with the control. The RLP in the groups treated with *P. putida* was higher than that in those treated with organic green. Although probiotics are an important management tool in aquaculture, it should be subjected to scientific laboratory tests and field measurements.

## 1. Introduction

Probiotics were firstly detected by Metchinkoff [1], who noticed that some acid-producing micro-organisms in fermented dairy products might prevent fouling in the intestine that led to a prolongation in the lifespan of humans. Today, probiotics are available in a variety of food products and have got wide applications in the control of cholesterol, cancers, and allergies [2] Lilley and Stillwell [3] mentioned that probiotics are substances secreted by a microorganism. Later on, probiotics were defined by several authors as microbial cell preparations that have a beneficial effect on the health and well-being of the host [4–6].

Probiotic was first recorded in fermented milk. After that, probiotics became popular with animal nutrition. Metchinkoff [1] suggested that people should consume fermented milk containing lactobacilli to prolong their lives. Accelerated aging is because of autointoxication (chronic toxemia), which is due to the toxins produced by gut microflora. The pathological reaction might be removed, and life expectancy could be enhanced by implanting lactic acid bacteria from yogurt [1]. Since then, researchers started investigations relating to the role of lactic acid bacteria in human and animal health.

Probiotics have been used in pigs as growth promoters [7], for lactose intolerance in rats, and antitumour and anticholestrolaemic effects in human [8, 9]. The main fields of research with respect to probiotics are heart diseases, allergic reaction, cancer, and diarrhoea. The use of probiotics results in alleviation of lactose intolerance [10], relief from constipation [11], and antitumour activities [12]. Intestinal infections caused by *Escherichia coli*, *Campylobacter fetus subsp. jejuni*, *Clostridium perfringens*, and *C. botulinum* were reduced in man and animal with the presence of *Lactobacillus* supplements [4]. *Bifidobacterium longum* has been successfully used to reduce the later effects of antibiotic therapy [13].

However, probiotics of aquatic sources could be endogenous or exogenous microbiota, and the isolated probiotics from the endogenous microbiota may depend on genetic, nutritional, and environmental factors. As the ambient environment has a greater influence on the health status of the aquatic animals than for terrestrial animals, human probiotics obtained from the aquatic species have a much larger influence on the health status [14].

Disease outbreaks are increasingly being recognized as a major constraint in aquaculture production and the economic development in many countries. Conventional approaches, such as the use of disinfectants and antimicrobial drugs, have had limited success in the prevention or the cure of aquatic disease. Bacterial diseases, especially due to *A. hydrophila*, are responsible for heavy mortality in fish. Antibiotics are used to control these infections but may develop and spread antimicrobial-resistant bacteria and resistance genes [15]. The sensitivity of *A. hydrophila* isolates to some antibiotics revealed a high sensitivity reaction to cefquinome [16]. In the last decade, Roman [17] mentioned that *A. hydrophila* infection in fish is sensitive to some of the fourth-generation cephalosporins including the cefipime. Furthermore, there is a growing concern about the use and, particularly, the abuse of antimicrobial drugs not only in human medicine and agriculture but also in aquaculture where this could induce hazard through the development of cross-resistance to antimicrobials used in human medicine [18]. Therefore, FDA's Center for Veterinary Medicine (CVM) regulates the manufacture, distribution, and use of animal's drugs. FDA also has established safe maximum residue limits (MRLs) for these drugs and other veterinary medications. Such strict regulations were taken to ensure that the treated animals are free from potentially harmful residues [19].

The concept of biological disease control has received widespread attention in the last decade; therefore, commercial probiotics are increasingly used in fish farming, but further investigations are required to identify the most suitable microbial preparations and doses for each fish species. Besides the high cost associated with purchasing a commercial product of probiotics, the variability in response to probiotics and the lack of reliable data hinder the use of such practices routinely in aquaculture [15]. An effective probiotic should be obtained from the same animal species. The underlying reason for this is that the intestines of individual species are sufficiently different from those of others, such that the isolates suited to those environments would not necessarily be suited to the intestine of others [15]. To ensure the required immunological response, alleviate the problem of introducing new microbial agents to our environment, and the high costs of commercial probiotics, isolation of native strains from native fish is important. For these reasons, the present study aimed to evaluate the effects of the isolated field strain of probiotics by determining their inhibitory effect against pathogenic *A. hydrophila* and evaluate their role in increasing the immune response, as well as the resistance of cultured tilapia fish to infection in comparison with other available commercial probiotics.

## 2. Materials and Methods

### 2.1. Fish

A total number of 250 live and apparently healthy Nile tilapia (*O. niloticus*) of both sexes were collected from Fish Research Institute and used in this study. Ten *O. niloticus* (60 ± 5 g) were used to isolate the probiotic, 90 *O. niloticus* (60 ± 5 g) were used for the safety experiment, and 180 *O. niloticus* (30 ± 10 g) were used in the feeding experiment. Fish were kept in fiber glass tanks containing dechlorinated tap water and supplied with continuous air, and feces was siphoned daily. Fish were fed twice daily with a balanced diet at a rate of 3% body weight and kept for two weeks under observation for acclimation.

### 2.2. Bacterial Isolation and Identification

**Figure 1:**
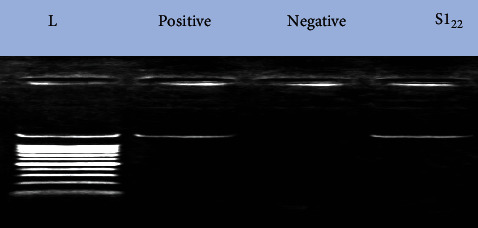
Gel electrophoresis of the PCR product of the isolated probiotic strain, where lane1, molecular weight ladder; lane 2, a positive control; lane 3, a negative control; and lane 4 (S1), the specific DNA product of about 1485 base pairs (bp) amplified from the isolate.

Ten *O. niloticus* (5 apparently healthy and 5 with disease signs, each 60 ± 5 g) were randomly collected from earthen ponds. Bacteriological examination of the fish samples was carried out. Swab samples were taken from the internal organs (liver, kidney, gonads, stomach, and intestine) and gills; they cultured on tryptic soya broth (TSB) and incubated at 30°C for 1 to 2 days. Pure isolates were taken after subculture on tryptic soya agar (TSA). Identification of the strain was performed using biochemical tests according to [20] and the API 20 *E* strip system (Bio Merieux), as well as the molecular technique (the PCR product of the isolated strain was used in gel electrophoresis (Figure 1) and 16S rRNA sequencing, and the strain was identified as *P.putida*). Pathogenic *A. hydrophila* strain was obtained as a reference strain.

### 2.3. Antimicrobial Activity Assay

The bacteria were tested for their probiotic activity in vitro using an agar spot assay [21]. The probiotic strain was cultivated in trypticase soya broth (TSB) (BioLife Milano, Italy) and incubated at 30 1°C for 24 h. Then, spots were made by pouring 10 mL of a well-grown overnight culture of the probiotic strains in the centre of the trypticase soya agar plates. The plates were incubated overnight at 30°C, and the growth of the strains was checked the next day. After the spots were developed, a soft agar (composed of tryptone soya broth10.7% bacteriological agar containing 5% of an overnight culture of pathogenic strains of *A. hydrophila* in tryptone soya broth) was poured on the plates. Inhibition was recorded by measuring the absence of pathogen growth around the spots.

### 2.4. Basal Diet

Pellets (0.5 cm) were prepared from locally available ingredients using a pellet machine (CPM California Pellet mill, San Francisco, CA, USA). The ingredients (Table 1) were mixed mechanically with a horizontal mixer (Hobartsmodel D300 T, Troy, OH, USA) at a low speed for 30 min after crushing the corn to a size of 0.5 mm using a Thomas-Willey Laboratory Mill Model 4. Then, oil was added gradually to ensure an even distribution of the ingredients with an increase in the mixer speed for 5 min, during the time when 600 mL water was added. The pellets obtained were allowed to air dry at room temperature for 24 h. The required diet was prepared biweekly and stored in a refrigerator (4 1°C) for daily use.

### 2.5. Preparation of Feed with Probiotics

Preparation of probiotic bacteria was carried out by inoculating the isolates *(P. Putada*) in TSB and incubating for 48 h at 30°C. They were then centrifuged at 3000 ×g for 30 min. After centrifugation, the bacteria were washed twice with sterile saline, and the concentration of the final suspension was adjusted to 1 × 10^10^ bacteria/ml in saline. The bacterial suspension containing the probiotic isolates was added to commercial food (containing 25% protein) to give 1 × 10^9^ bacterial cells/g of diet for the viability experiment and 1 × 10^7^ bacterial cells/g of diet for the feeding experiment, by mixing well with an automatic mixer. The pellets were dried in an oven at 45°C. To determine the viability of the probiotics, one half of the feed was stored in a refrigerator (4°C) while the other half was kept at 25 ± 1°C. For the feeding experiment, the feed was stored in a refrigerator at 4°C.

### 2.6. Organic Green^™^

Organic Green™ is a commercial product available in the market and manufactured by Hang Poong Industry, Inchon City, Korea. It is used to improve the growth and resistance of poultry and large animals. It is a mixture of probiotics, 1 kg of this product containing 1 × 10^11^ bacterial cells each from *Lactobacillus acidophilus, Bacillus subtilis, Saccharomyces*, and *Aspergillus oryzae* according to the manufacturers. This commercial product was tested in the laboratory for isolation and identification of viable organisms. One dose of 1 g of Organic Green™/kg feed was mixed, and pellets were made. The pellets were prepared biweekly, air-dried at room temperature for 24 h, and stored in a refrigerator (4°C).

### 2.7. Safety of the Tested Probiotic Strains

Ninety tilapia (65 ± 5 g) were divided into 2 equal groups, each with three replicates (each with 15 fish) and distributed randomly among 6 aquaria. The first group was intraperitoneally (I/P) injected with 0.5 ml L acidophilus fresh culture suspension containing 10^7^ bacteria/ml, while the second group served as a control and was I/P injected 0.5 ml sterile saline (0.85% NaCl). Both the test and control group of fish were observed and fed on a basal diet containing 30% protein and water temperature was 26°C throughout the experiment. The mortality rate was recorded daily for 15 days.

### 2.8. Experimental Design

One hundred and eighty Nile tilapia with an average body weight (30 ± 10 g) were divided into 3 equal groups, each with 30 fish. Each group was divided equally into 3 replicates (10 fish per each). The fish were acclimated in in-door fiberglass tanks for 14 days. Each tank was supplied with a well-oxygenated tap water. Group (1): the control was fed basal diet without bacteria, group (2): fed basal diet containing *P. Putida*, at a dose of 1 × 10^8^ CFU/g, and group (3): fed basal diet with Organic Green™ 1.0 g/kg diet.The prepared diet was transferred to plastic bags and stored in a refrigerator (4°C), and this preparation was repeated every two weeks. Fish were fed 6 days a week for 60 days. The dead fish were recorded and removed daily.

### 2.9. Blood Sampling

Twenty fish were randomly collected from each treatment and the control. The fish were anesthetized by immersion in water containing 0.1 ppm tricaine methane sulfonate (MS-222). Whole blood (0.5 ml) was collected from the caudal vein of each fish using syringes (1-ml) and 27-gauge needles that were rinsed in heparin (15 unit/ml), to determine the hematocrit values, NBT, and phagocytic activity tests. A further 0.5 ml blood sample was centrifuged at 1000 ×g for 5 min in order to separate the plasma. The latter was stored at −20°C to be used for the lysozyme activity test. For separation of serum, blood samples (0.5 ml) were withdrawn from the fish caudal vein, as before, and transferred to Eppendorf tubes without an anticoagulant. The blood samples were centrifuged at 3000 ×g for 15 min, and the supernatant serum was collected and stored at −20°C until used for the serum bactericidal test [22].

### 2.10. Hematological and Immunological Parameters

#### 2.10.1. Hematology Parameters

Hematocrit capillary tubes were two-third filled with the whole blood and centrifuged in a hematocrit centrifuge for 5 min, and the percentage of the packed cell-volume was determined by using the hematocrit tube reader. The WBC count was determined by using a Neubauer hemocytometer [23, 24]. Blood was diluted 1 : 20 with Turk's diluting fluid and placed in a hemocytometer. Four large (1 sq mm) corner squares of the hemocytometer were counted under the microscope. The cells touching the boundary lines were not counted. The total number of WBC was calculated in mm^3^ × 103 [25]. The blood smears were prepared and stained with Giemsa stain for 30 min. One hundred leukocytes were identified, and the percentage values of different white cells were calculated according to [26].

#### 2.10.2. Nitroblue Tetrazolium Activity (NBT)

Blood (0.1 ml) was placed in microtiter plate wells, where an equal amount of 0.2% NBT solution was added and incubated at room temperature for 30 min. N dimethyl formamide 1 ml was added to a sample of NBT blood cell suspension (0.05 ml) in a glass tube and centrifuged at 3000 rpm for 5 min. The supernatant was measured using a spectrophotometer at 620 nm in 1 ml cuvettes [22, 24].

#### 2.10.3. Lysozyme Activity

Chicken egg lysozyme (Sigma) was the standard, and 0.2 mg/ml *Micrococcus lysodeikticus* (lyophilized form) in 0.04 M sodium phosphate buffer (pH 5.75) was used as the substrate. Fifty *µ*l of serum was added to 2 ml bacterial suspension, and the reduction in the absorbance at 540 nm was determined after 0.5 and 4.5 min incubation at 22°C [27].

#### 2.10.4. Phagocytic Activity

Phagocytosis assay was performed according to the method described by [28] with some modifications. One ml of the adjusted viable leukocytes suspension (leukocytes in RPMI1640 with 5% of pooled tilapia serum) was placed in sterile plastic tube, to which 1 ml of the prepared heat in activated *C.Glabrata* was added. The tubes were then incubated for 30 min at 27°C in a 5% CO_2_ incubator. Then, the tubes were centrifuged at 2500 rpm for 5 min, and the supernatant was removed. Slide smears were prepared from the deposit, air dried, and then stained with Leishman's stain.

### 2.11. Histopathological Examination

Tissue specimens including the liver, kidney, spleen, and intestine from each experimental group of treatments were collected by the end of feeding experiment (2 months). The collected specimens were immediately fixed in neutral buffered formalin 10%, dehydrated in ascending concentration of ethyl alcohol, cleared in two changes of xylene, blocked in paraffin, and sectioned at 5 *µ*m using a rotary microtome. The microscopic tissue slides were stained with routine hematoxylin and eosin stain (H&E stain) and then covered with cover slips. The histopathological technique was performed according to Wallington (1980).

### 2.12. Response to Challenge Infections

Thirty fish from each of the tested treatments (10 from each replicate) were collected and reared in a glass aquarium. They were clinically examined, and blood samples were bacteriologically tested and proved to be free from bacterial infection. The treatment groups were subjected to challenge infections, after feeding with test diets for 1 month (15 fish for the first phase) and 2 months (15 fish for the second phase). The challenged bacteria were obtained as a reference pathogenic strain of *A. hydrophila* that were isolated previously from the liver of morbid *O. niloticus* and studied for pathogenicity.

A culture suspension of *A. hydrophila* was prepared by culturing in agar for 24 h, collected, washed and suspended in sterile saline 0.85%, and counted using Mc Firland standard tubes. Then, fish were artificially infected by an intraperitoneal injection with 0.5 mL of culture suspension of pathogenic *A. hydrophila* containing 10^8^ bacteria/L. The relative level of protection (RLP) among the challenged fish was determined [22, 29] using the following equation: RLP(1/4)100 − percent of immunized mortality/percent of control mortality × 100.

### 2.13. Statistical Analysis

Analysis was performed to the measured growth and immunological parameters of the collected samples using the analysis of variance (ANOVA) and Duncan's multiple range test [30] (mean at a significance level of *P* < 0.05). Analysis was performed using Minitab (18) package.

## 3. Results

### 3.1. Isolation and Identification of the Probiotic Isolates

Five bacterial isolates were obtained from the intestinal tract of 10 fish (*O. niloticus*). The five isolates were investigated for their inhibitory activity against pathogenic *A.hydrophila*. Only two isolates showed an inhibitory effect against *A.hydrophila*. The inhibition zones were 15 and16 mm in diameter. The isolate that showed the largest inhibition zone (16 mm) in the in vitro antimicrobial activity test was preliminary identified as *P.putida* using (API20 E) strips with code 2204046. The PCR product of the isolated strain was used in gel electrophoresis (Figure 1) and 16S rRNA sequencing, and the strain was identified as *P.putida.*

### 3.2. Safety and Survival Rate

The intraperitoneal injection of *O. niloticus* with *P. putida* at a dose of 0.3 ml matching 3 × 10^7^ CFU/ml was noticed to be safe, as well as causing no mortalities during a period of 15 days, indicating the safety of the isolated bacterial strain. During the first and the second month of experiment, there was a highly significant increase in the survival percent of the experimental fish in both treated groups with probiotics (the first group fed *P.putida* and the second group received Organic Green™) when compared with the control group (Table 2).

### 3.3. Hematological and Immunological Parameters

The first phase of the experiment where the fish were given a basal diet mixed with probiotics for 1 month revealed a significant increase in the hematocrit values in all treated groups in comparison with the control. A significant increase in NBT, lysozyme activity, and phagocytic activity was seen in all treated groups in comparison with the control. The increase in the TLC was significant in the group fed with *P. putida* in comparison with the control group. Although the number of neutrophils had nonsignificantly increased in *P. putida*-treated groups in comparison with the control, the increase in TLC resulted mainly from the increase in lymphocytes and monocytes (Table 2).

In the second phase, the fish which were given a basal diet mixed with probiotics for 2 months revealed a nonsignificant increase in the hematocrit values. Significant increases in the NBT and phagocytic index were seen in *P. putida* and organic green groups in comparison with the control group. The TLC and DLC revealed nonsignificant changes in the treated groups in comparison with the control (Table 2).

### 3.4. Relative Level of Protection of *O. niloticus* after Bacterial Challenge with *A. hydrophila*

The RLP in the groups treated with *P. putida* was higher than that in those treated with Organic Green™ (Table 2).

### 3.5. Histopathological Findings

The control group showed normal cellular details and tissue architecture with no marked degenerative changes or inflammatory reactions.

#### 3.5.1. Tilapia Received Basal Diet Mixed with *P. putida* at a Dose of 1 × 10^8^ CFU/ml for 2 Months

**Figure 2:**
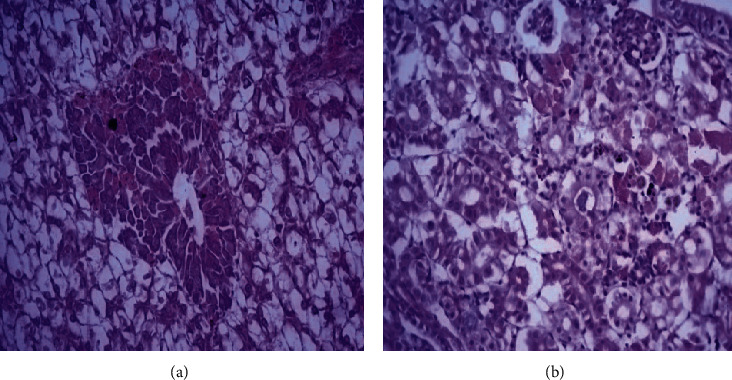
Histopathological findings of Tilapia treated with strain 1 *P. putida* with dose 1 × 10^8^ CFU/ml for 2 months showing (a) hepatic cellular vacuolation and focal proliferation of MMC and (b) mild tubular nephrosis in the form of vacuolar degeneration in the renal epithelium with mild interstitial edema and focal proliferation with leukocytes together with focal activation of melanomacrophage centers (H&E stain, a,b,d × 400,c × 100).

The liver and kidney revealed mild vacuolar degeneration in the hepatocytes and renal epithelium. Focal proliferation of melanomacrophage cells was evident in the hepatopancreatic and renal tissues. The renal interstitial issue showed a mild edema and focal proliferation with leukocytes. The spleen showed congestion in blood vessels with focal proliferation of lymphocytes and mild proliferation of melanomacrophage centers (Figures 2(a) and 2(b)).

#### 3.5.2. Tilapia Received Basal Diet Mixed with 1 gm/kg Diet Organic Green^™^ for 2 Months

**Figure 3:**
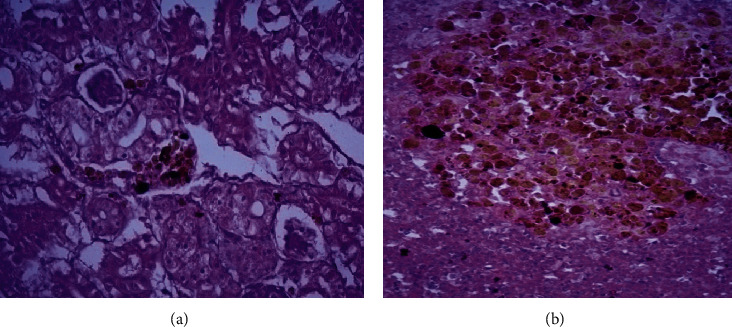
Histopathological findings of Tilapia treated with Organic Green™ with a dose 1 g/kg diet for 2 months showing (a) minimal degenerative changes in renal tissue, massive proliferation of melanomacrophage centers compared to other groups, and (b) splenic massive proliferation and activation of melanomacrophage centers (H&E stain, ×400).

The hepatopancreas and kidney exhibited minimal degenerative changes with aggregation of melanomacrophage cells around hepatopancreatic areas and in the renal parenchyma. The spleen revealed massive proliferation and activation of melanomacrophage centers all over the splenic parenchyma (Figures 3(a) and 3(b)).

## 4. Discussion

Currently, probiotics are available in a several food products, exclusively dairy products, due to the historical association of lactic acid bacteria with fermented milk. Probiotics are gaining importance for multiple benefits, e.g., treating lactose intolerance, hypercholesterol problem, cardiac diseases, and managing cardiac problems such as atherosclerosis and arteriosclerosis. Many probiotic products are present in the market place, supporting the evidence of health claims. New legislation governing the labelling of probiotics, such as indicating the species, strain, and number of bacteria present, is likely to come into force in the near future. Probiotics can be incorporated into a balanced diet to maximize good health. The main characteristics of microbes as candidate probiotics are to improve the health of their host, to antagonize pathogens, to have a colonization potential, and to be efficient in increasing the resistance to disease of their host. Gatesoupe [31] reported other beneficial effects of probiotics, e.g., competition with pathogens for nutrients or for adhesion sites and stimulation of the immune system. However, although probiotics may display multiple effects, possibly combining bacterial antagonism to some effects on the host, e.g., stimulating immunity or growth [32], competitive exclusion, enzyme activator [33], hormones inhibitor [34], immune response enhancement [35], and their modes of action, however, are not fully understood.

It is very important to characterize and identify the mode of action of the potential probiotics and their efficiency on the pathogen and safety. This can be achieved through in vitro and in vivo studies. The selection criteria of Gomez-Gil et al. [2] are based on the collection of background information; acquisition of potential probiotics; evaluation of the ability to outcompete pathogenic strains; and assessment of their pathogenicity and effect on the host; as well as economic analysis.

The United Nations has recommended some specifications to be considered when a probiotic is selected and approved [36] including viability of the probiotic to survive; colonization; competition against pathogenic bacteria; inhibiting pathogenic bacteria; resistance against other sanitary agents or disinfectants; and labelling according to the international nomenclature including dosing and the expiration date.

Bacterial diseases are responsible for heavy mortality in fish. Antibiotics are used to control these infections but may develop and spread antimicrobial-resistant bacteria and resistance genes [15]. As a consequence, prevention of fish disease by the application of live pathogen-antagonistic bacteria has received a widespread interest [37]. In the present study, samples from the intestine were cultured on TSB and incubated at 30°C for 24–48h. The isolated strains were purified through subculturing on TSA. Twelve bacterial isolates were obtained from the intestinal tract of tilapia fish (*O.niloticus*), and only two isolates showed an inhibitory effect against the pathogenic A. hydrophila. The isolate of the largest zone was identified as *P.putida* using API20 E and further molecular diagnostic tools. Similar findings were reported by Sebastião et al. [37] who isolated both *P. putida* (27%) from the spleen of tilapia fish and *P. fulva* (20%) from the skin and the kidney.

Abdel-Galil Ahmed et al. [38] also isolated many probiotic strains (*Vibrio* and *Pseudomonas* sp.) from endogenous and exogenous microbiota of a variety of species of marine fish. In our study, the antimicrobial activity assay was performed by applying agar disc diffusion method. The inhibition zones of isolated probiotics were 15 and 16 mm against pathogenic *A. hydrophila*. Related results were reported by Aly et al. [22, 39] who recorded that *B. subtilis* and *L. acidophilus* inhibited the growth of *A. hydrophila* in vitro. Similar findings were also detected by Abdl El-Rhman et al. [40] who isolated and identified both *M. luteus* and *Pseudomonas* sp., after isolation from the intestine and gonads of Nile tilapia *O.niloticus*, and reported *M. luteus* and Ps.

The intraperitoneal injection of *O.niloticus* with *P. putida* at a dose of 0.3 ml matching 3 × 10^7^ CFU/ml was noticed to be safe, as well as causing no mortalities during a period of 15 days, indicating the safety of the isolated bacterial strain. This result of safety experiment revealed no mortalities after an intraperitoneal injection of *O.niloticus* with *P. putida* at a dose of 0.3 ml matching 3 × 10^7^ CFU/ml during a period of 15 days, which was supported by Eissa and Abou [41] who tested the isolated *P. fluroscens* biovars I, II, and III and noticed that they were nonpathogenic and safe to *O. niloticus*.

The first phase of the experiment revealed a significant increase in the hematocrit values in all treated groups together with an increase in the TLC that was significant in the group fed with *P. putida* in comparison with the control group. Aly et al. [22, 42] proved that *B. pumilus* significantly increases the NBT values, hematocrit values, total leucocytic, and differential leucocytic count, with a significant increase in lymphocytes and monocytes in *O. niloticus*. Sakai et al. [35] mentioned that the nonspecific immune system of the fish can be stimulated by probiotics. Lysozyme has a bactericidal effect by destroying cellular walls of bacteria. Lysozyme also stimulates the phagocytic activity and participates in the regulation of immune cell differentiation and proliferation [43]. Fish Phagocytic activity represents an immediate response carried out by the phagocytes to kill the pathogenic bacteria as a part of their defense mechanism [44]. It is a key element in host defenses against bacterial infections (Kantari et al. 2008). Concerning the measured immunological parameters in the present study, the results of NBT, lysozyme activity, and phagocytic activity after one month of experiment showed a significant increase in the groups which received probiotics in relation to the control and could be attributed to the increased blood and immunological parameter. Aly et al. [22] reported that *B. subtilis* and *L. acidophilus* significantly increase the nitroblue-tetrazolium (NBT) assay, neutrophil adherence, and lysozyme activity and showed a significant increase in the serum bactericidal activity in *O. niloticus*.

The relative level of protection (RLP) of Nile tilapia-treated groups with *P.putida* or organic green after challenging with *A. hydrophila* through the 1st and 2nd month of experiment was 62.5, 55.5, 52.5, and 44.4, respectively. These results agreed with those of Aly et al. [22] who demonstrated that the relative level of protection (RLP) against *P. fluorescens* and *A. hydrophila* was significantly elevated in Nile tilapia fed on *B. subtilis* and *Lactobacillus*. Gram et al. [45] also recorded that *P. fluorescens* AH2 protects rainbow trout against challenge with *V. anguillarum*. Li and Gatlin [46] also found that yeast (GrostBioticR-A) protects hybrid striped bass against mycobacterial infection.

## 5. Conclusions

The concept of biological disease control using probiotics has received a widespread attention during the last decade for their cheaper value and more safety than antibiotics. In order to define the potential of probiotics in aquaculture, selection criteria are crucial. Data about the efficiency, mode of action, safety, durability, and economic cost benefit should be known. Although probiotics are an important management tool in aquaculture as an alternative to antimicrobials use and to the shortage in the vaccine availability, they should be subjected to scientific laboratory tests and field and economic measurements to be recommended in a large-scale aquaculture.

## Figures and Tables

**Table 1:** Composition of the basal diet used throughout the experiment.

Ingredients	Diet (%)
Fish meal	7.85
Soybean meal	52.9
Ground corn	29.1
Wheat flower	5.00
Vegetable oil	2.00
Cod liver oil	2.00
Dicalcium phosphate	1.00
Mineral mix	0.07
Vitamin mix	0.05
Total	100

**Table 2:** Survival, immunological parameters, and the relative level of protection of the experimental groups after one and two months of feeding experiment.

Parameter	Control		*P. putida*		Organic Green™	
	1st month	2nd month	1st month	2nd month	1st month	2nd month
Survival %	92.55 ± 1.98^b^	92.64 ± 0.10^b^	98.12 ± 0.91^a^	98.12 ± 0.96^a^	96.23 ± 0.91^a^	96.29 ± 0.96^a^
Hematocrit (%)	30.6^A^ ± 2.09	30.2^A^ ± 1.81	31.0^A^ ± 1.89	29.6^A^ ± 1.62	31.0^A^ ± 0.98	28.8^A^ ± 1.03
TLC (×10^3^/mL)	36.5^BC^ ± 1.45	40.2^A^ ± 0.99	40.2^A^ ± 0.84	37.6^AB^ ± 1.09	33.4^C^ ± 1.49	37.6^AB^ ± 1.09
Neutrophils	11.85^A^ ± 0.15	12.14^A^ ± 0.19	12.03^A^ ± 0.24	12.18^A^ ± 0.16	11.23^B^ ± 0.15	11.35^AB^ ± 0.14
Lymphocytes	23.4^BC^ ± 1.14	26.53^A^ ± 0.84	26.04^A^ ± 1.09	25.22^A^ ± 0.64	21.11^C^ ± 0.69	24.62^AB^ ± 0.91
Monocytes	0.90^B^ ± 0.12	0.92^A^ ± 0.13	1.41^A^ ± 0.16	1.12^A^ ± 0.14	0.98^B^ ± 0.17	0.95^A^ ± 0.14
Eosinophils	0.31^A^ ± 0.08	0.53^A^ ± 0.07	0.33^A^ ± 0.05	0.32^B^ ± 0.05	0.28^A^ ± 0.05	0.6^A^ ± 0.08
Basophils	0.04^A^ ± 0.04	0.83^A^ ± 0.06	0.11^A^ ± 0.06	0.12^A^ ± 0.06	0.12^A^ ± 0.06	0.09^A^ ± 0.06
NBT mg/ml	0.06 ± 0.01^b^	0.07 ± 0.01^b^	0.27 ± 0.01^a^	0.25 ± 0.01^a^	0.24 ± 0.00^a^	0.22 ± 0.00^a^
Lysozyme activity unit/ml	0.72 ± 0.05^b^	1.22 ± 0.03^b^	1.60 ± 0.12^a^	1.84 ± 0.09^ab^	1.68 ± 0.12^a^	1.43 ± 0.03^b^
Phagocytic index	1.30 ± 0.03^b^	1.40 ± 0.01^b^	1.60 ± 0.02^a^	1.90 ± 0.02^a^	1.67 ± 0.02^a^	1.97 ± 0.03^a^
Phagocytic %	22.70 ± 0.01^b^	22.60 ± 0.01^b^	28.90 ± 0.04^ab^	30.20 ± 0.04^a^	33.20 ± 0.05^a^	34.20 ± 0.05^a^
RLP (%)	0.0	0.0	62.5	55.5	52.5	44.4

Mean ± SE having the same letter in the same row are not significantly different at *P* < 0.05.

## Data Availability

All required data are included in tables and figures.
